# Dietary Glucose Ameliorates Impaired Intestinal Development and Immune Homeostasis Disorders Induced by Chronic Cold Stress in Pig Model

**DOI:** 10.3390/ijms23147730

**Published:** 2022-07-13

**Authors:** Guodong Sun, Xin Song, Yingbin Zou, Teng Teng, Lin Jiang, Baoming Shi

**Affiliations:** Institute of Animal Nutrition, Northeast Agricultural University, Harbin 150030, China; s976031645@163.com (G.S.); xinsongs@163.com (X.S.); zyb19961102@163.com (Y.Z.); b20050115@neau.edu.cn (T.T.); jianglin0046@163.com (L.J.)

**Keywords:** chronic cold stress, small intestinal mucosa, TLR4/MyD88 signal pathway, pyroptosis, glucose

## Abstract

Endotherms are easily challenged by chronic cold stress. In this study, the development and injury of the small intestine in the Min pig model and Yorkshire pig model under chronic cold stress, and the molecular mechanisms by which glucose supplementation reduces small intestinal mucosal damage were investigated. The results showed that morphological structure lesions of the jejunal mucosa and ileal mucosa were visible in Yorkshire pigs under chronic cold stress. Meanwhile, the Occludin mRNA and protein expression in jejunal mucosa of Yorkshire pigs was decreased. Chronic cold stress enhanced the expression of Toll-like receptor 4 (TLR4), the myeloid differentiation main response 88 (MyD88), nucleotide-binding domain and leucine-rich repeat protein 3 (NLRP3), cleaved caspase-1, mature-IL-1β, and high-mobility group box 1 (HMGB 1) mRNA and protein expression in jejunal mucosa of Yorkshire pigs, whereas the mRNA and protein of Bax was triggered in ileal mucosa. In Min pigs, no such deleterious consequences were observed. Dietary glucose supplementation ameliorates small intestinal mucosal injury, declined TLR4 and MyD88 expression in jejunal mucosa. In conclusion, chronic cold stress induced the small intestinal mucosa damage in Yorkshire pigs, whereas glucose supplementation mitigated the deleterious effects of chronic cold stress on the small intestine.

## 1. Introduction

Cold is a severe environmental challenge that endotherms have to deal with. The normal growth of animals is hampered, metabolic rate is altered, and the endocrine system is disrupted when endotherms are exposed to extreme or chronic cold stress [1,2,3]. Variations in ambient temperature have an impact on non-specific physiological changes in the immune system that may reduce the animal’s resistance to disease [4]. Some studies in piglet models have shown that chronic cold stress induces diarrhea [5], suggesting that chronic cold stress may disrupt the immune system of the intestinal mucosa, but the mechanism is not clear. Min pigs, a traditional native pig breed with low mortality in northeast China, have better cold adaptability [6,7]. In addition, Min pigs have stronger immune systems [8,9,10]. Although the cold adaptability of Min pigs is well known, little research has been carried out on the development of the small intestinal mucosa under chronic cold stress.

TLR4 has been extensively studied as a regulator of inflammation and tissue damage. TLR4 can recruit MyD88 to trigger nuclear factor-κB (NF-κB) and promote the transcription of inflammatory factors [11,12]; TLR4 also recognizes lipopolysaccharide and induce pro-inflammatory reaction [13]. Furthermore, overexpression of TLR4 has been demonstrated to worsen tissue damage and inflammation [14]. In vivo, cold stress has been proven to activate the TLR4 pathway [15], resulting in tissue damage, but little research exists on the small intestinal mucosa. Pyroptosis is a novel type of programmed cell death that involves the production of pro-inflammatory cytokines and relies on inflammatory caspases (e.g., caspase-1) [16]. To maintain the intracellular environment and better adapt to the survival environment, mammals will trigger cells to die autonomously and methodically through apoptosis [17]. Organisms regulate apoptosis by modulating the levels of pro-apoptotic and anti-apoptotic members of the B-cell lymphoma 2 (Bcl-2) family [18]. Disturbances in the apoptotic process may contribute to the development of a variety of diseases [19]. Chronic cold stress has been found to disrupt the balance of apoptosis and impair organ functions [20]. The effects of continuous cold stress on apoptotic pathways in the small intestine remain unclear during chronic cold stress.

Glucose is a quick source of energy. Starch, lactose, and sucrose in diets eventually are broken down into monosaccharides (such as glucose) in the small intestine [21]. Sodium-glucose cotransporter 1 (SGLT1) and GLUT-2 on intestinal mucosal cells transport glucose from the intestine to the blood and distribute it to various tissues and organs of the body for usage [22]. The small intestine also regulates appetite and pancreatic hormone secretion through the release of intestinal hormones, which in turn promote efficient energy absorption and storage [23]. The effect of glucose on the development of small intestinal mucosa under chronic cold stress has been little studied, although the mechanism of glucose absorption in the intestine has been intensively studied in mammals for a long time.

Cold is a common stressor for endotherms. Here, we explored the effects of chronic cold stress on intestinal mucosal development and intestinal immune homeostasis via establishing a cold adaptation model (Min pigs) and non-cold adaptation model (Yorkshire pigs). Furthermore, we explored the mechanism by which dietary glucose alleviates intestinal injury in cold-exposed pigs. Collectively, this study provides new evidence and interventions for the negative effects of chronic cold stress.

## 2. Results

### 2.1. Chronic Cold Stress Disrupts Intestinal Development, Intestinal Permeability, Physical Barriers and Intestinal Function in Pigs

**Figure 1:**
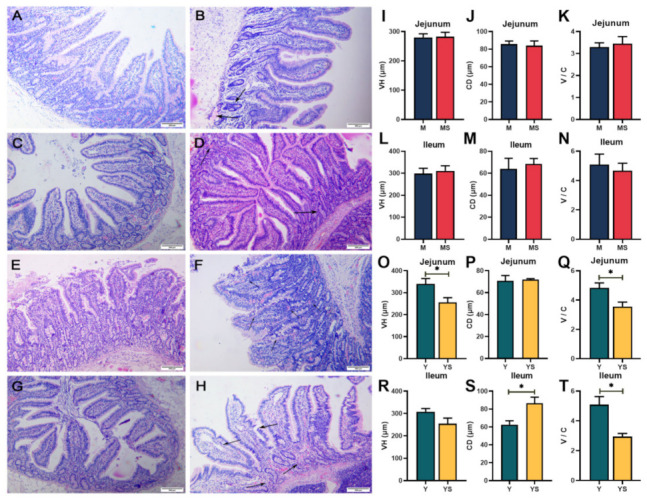
Effects of chronic cold stress on jejunum and ileum slices in pigs. (**A**–**D**) Chronic cold stress did not induce considerable harm to the jejunal mucosa and ileal mucosa of the Min pigs. (**E**) In the jejunal mucosa of the Yorkshire pigs in the Y group, no major damage was discovered. (**F**) In the YS group, the jejunal mucosa had more inflammatory changes and multiple hemorrhages, and crypt and villi were reduced. (**G**) No obvious impairment of the ileal mucosa was observed in the Yorkshire pigs of the Y group. (**H**) In the YS group, the ileal mucosa was edematous, with epithelial rupture, inflammatory cell infiltration and reduced goblet cells, and mucosal hemorrhage was visible. (**I**–**N**) VH, CD, and V/C in jejunum and ileum of Min pigs. (**O**–**T**) VH, CD, and V/C in jejunum and ileum of Yorkshire pigs. Data are presented as the means ± SEM. * *p <* 0.05.

The evaluation of pathological sections of the jejunal mucosa and ileal mucosa is shown in Figure 1. The jejunum mucosa was largely intact in both the Min control group (M group) and Min cold stress group (MS group), the mucosal folds were apparent, and the goblet cells were essentially normal (Figure 1A,B). The jejunal mucosa of the Yorkshire cold stress group (YS group) showed more inflammatory changes and multiple hemorrhagic spots were seen, and the goblet cells, crypt, and villi were reduced (Figure 1F). The mucosal surface of the ileal mucosa was relatively intact in the M and MS groups, with visible mucosal folds, and a few inflammatory changes in the mucosa (Figure 1C,D). The mucosa of ileal mucosa in the Yorkshire control group (Y group) had few inflammatory changes, and the morphology of glands, goblet cells, crypts, and villi were basically normal (Figure 1G). The YS group showed ileal mucosal edema, punctate epithelial disruption, partial infiltration of inflammatory cells, and reduction of goblet cells (Figure 1H). As shown in Figure 1, under the influence of chronic cold stress, there were no significant changes in villus height (VH), crypt depth (CD), and VH/CD (V/C) of the jejunum and ileum in the MS group compared with the M group (*p >* 0.05). Compared with the Y group, chronic cold stress had no effect on CD in the jejunum and VH in the ileum in the YS group (*p >* 0.05, Figure 1P,R). Furthermore, in the YS group, VH of the jejunum (*p <* 0.05, Figure 1O) and V/C of jejunum and ileum were considerably lower than in the Y group (*p <* 0.05, Figure 1Q,T), while the CD of ileum was significantly higher than that in the Y group (*p <* 0.05, Figure 1S).

**Figure 2:**
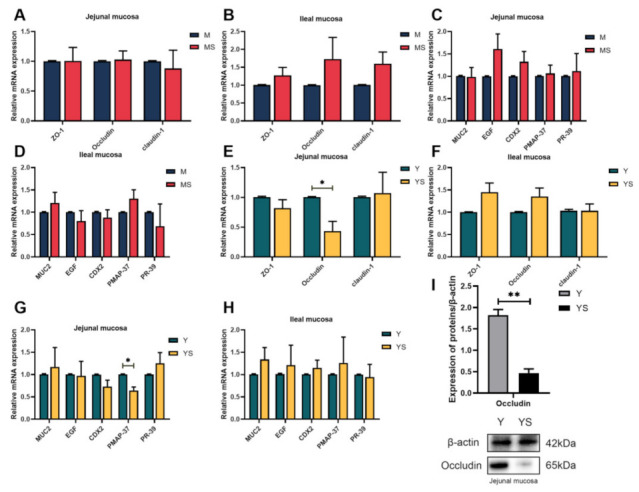
Effects of chronic cold stress on intestinal growth and development of pigs. (**A**,**B**) The mRNA expression levels of tight junction proteins in the intestinal mucosa of Min pigs (n = 6). (**C**,**D**) The mRNA expression levels of developmental function-related genes in the intestinal mucosa of Min pigs (n = 6). (**E**,**F**) The mRNA expression levels of tight-junction proteins in the intestinal mucosa of Yorkshire pigs (n = 6). (**G**,**H**) The mRNA expression levels of developmental function-related genes in the intestinal mucosa of Yorkshire pigs (n = 6). (**I**) The protein expression level of Occludin in the jejunal mucosa of Yorkshire pigs (n = 6). Data are presented as the means ± SEM. * *p <* 0.05, ** *p <* 0.01.

To examine the effect of chronic cold stress on the physical barrier of jejunum and ileum, ZO-1, Occludin and claudin-1 were analyzed in the small intestinal mucosa (Figure 2). Chronic cold stress had no restrictive effect on the mucosal physical barrier of jejunum and ileum in Min pigs (*p >* 0.05, Figure 2A,B). However, in the YS group, the mRNA expression and protein expression of Occludin in jejunum mucosa was significantly diminished by chronic cold stress (*p <* 0.05, Figure 2E,I). As can be seen from Figure 2, MUC2, EGF and CDX2 were not significantly changed in jejunum and ileum, neither in experiment 1 nor experiment 2 (*p >* 0.05). Meanwhile, compared with the Y group, the mRNA expression of PMAP-37 in the jejunum of the YS group was decreased (*p <* 0.05, Figure 2G), but there was no remarkable change in PR-39 (*p >* 0.05, Figure 2G).

### 2.2. Chronic Cold Stress Increases the Risk of Inflammation in the Small Intestinal Mucosa of Pigs

**Figure 3:**
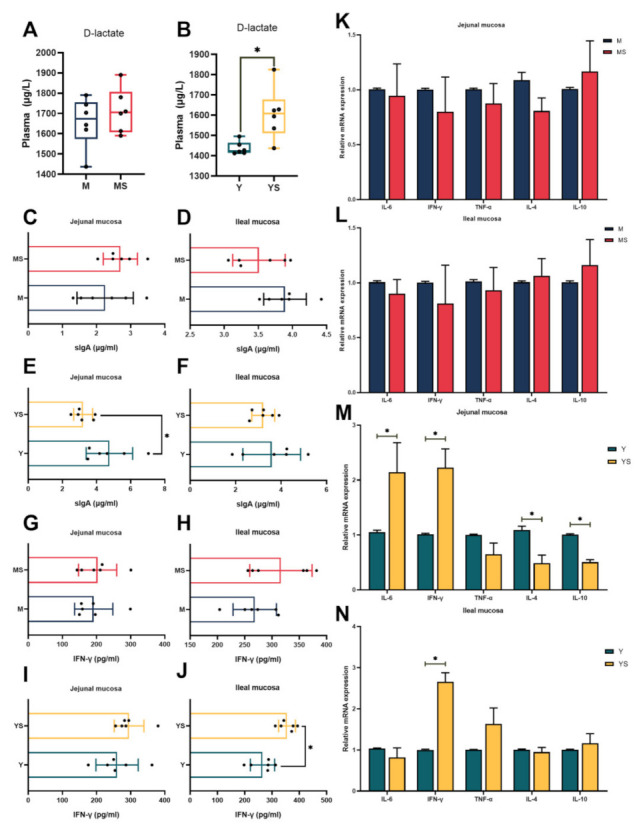
Effects of chronic cold stress on intestinal damage in pigs. (**A**) The level of D-lactate in the plasma of Min pigs (n = 6). (**B**) The level of D-lactate in the plasma of Yorkshire pigs (n = 6). (**C**,**D**) The content of sIgA in the intestinal mucosa of Min pigs (n = 6). (**E**,**F**) The content of sIgA in the intestinal mucosa of Yorkshire pigs (n = 6). (**G**,**H**) The content of IFN-γ in the intestinal mucosa of Min pigs (n = 6). (**I**,**J**) The content of IFN-γ in the intestinal mucosa of Yorkshire pigs (n = 6). (**K**,**L**) The mRNA expression levels of inflammatory factors in Min porcine intestinal mucosa (n = 6). (**M**,**N**) The mRNA expression levels of inflammatory factors in Yorkshire porcine intestinal mucosa (n = 6). Data are presented as the means ± SEM. * *p <* 0.05.

We next sought to identify the damaging effects of chronic cold stress on the jejunal mucosa and ileal mucosa. As shown in Figure 3B, the YS group had significantly higher (*p <* 0.05) plasma D-lactate activity than the Y groups. In experiment two, it was detected that the concentration of sIgA in the jejunal mucosa of the YS group appreciably dropped more than that of the Y group (*p <* 0.05, Figure 3E). Compared with the Y group, the mRNA expression of IL-6 and IFN-γ in jejunal mucosa of the YS group were up-regulated (*p <* 0.05, Figure 3M), while the mRNA expression levels of IL-4 and IL-10 were down-regulated (*p <* 0.05, Figure 3M). The mRNA expression level of IFN-γ in ileum mucosa of the YS group was significantly increased (*p <* 0.05, Figure 3N), and the protein concentration of IFN-γ was also increased (*p <* 0.05, Figure 3J) when compared with the Y group.

### 2.3. Chronic Cold Stress Induces Small Intestinal Mucosal Injury by Modulating TLR4/MyD88 Signaling Pathway

**Figure 4:**
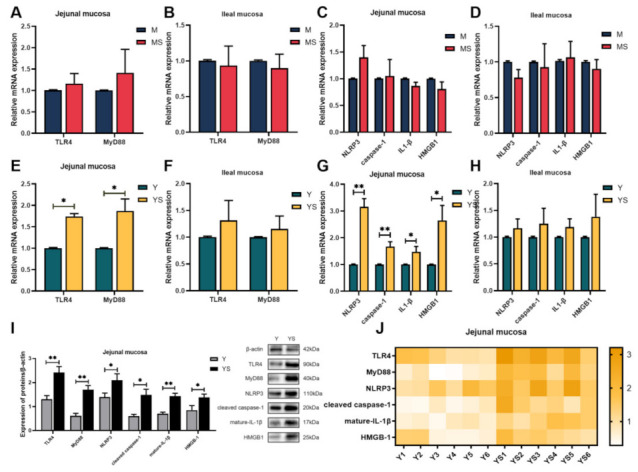
Effects of chronic cold stress on the expression of key genes and proteins in the TLR4/NLRP3 signaling pathway in the intestinal mucosa of pigs. (**A**,**B**) The mRNA expression of TLR4 and MyD88 in intestinal mucosa of Min pigs (n = 6). (**C**,**D**) The mRNA expression of key genes of pyroptosis in the intestinal mucosa of Min pigs (n = 6). (**E**,**F**) The mRNA expression of TLR4 and MyD88 in intestinal mucosa of Yorkshire pigs (n = 6). (**G**,**H**) The mRNA expression of key genes of pyroptosis in the intestinal mucosa of Yorkshire pigs (n = 6). (**I**,**J**) The expression of TLR4/NLRP3 signaling pathway-related proteins in the jejunal mucosa of Yorkshire pigs (n = 6). Data are presented as the means ± SEM. * *p <* 0.05, ** *p <* 0.01.

In order to determine whether chronic cold stress caused collective injury through the TLR4/MyD88 signal pathway, we analyzed the expression of related genes. From Figure 4, compared with the Y group, the mRNA and protein expression levels of TLR4 and MyD88 in jejunum mucosa of the YS group was considerably increased (*p* *<* 0.05, Figure 4E,I). In addition, the mRNA and protein expression levels of NLRP3, IL-1β, and caspase-1 were also considerably heightened (*p* *<* 0.05, Figure 4G,I). In this study, the mRNA and protein expression levels of HMGB 1 in jejunal mucosa in the YS group were significantly increased compared with the Y group (*p* *<* 0.05, Figure 4G,I). In contrast, no similar changes occurred in the Min pigs.

### 2.4. Chronic Cold Stress Induces Small Intestinal Mucosal Injury by Modulating TLR4/MyD88 Signaling Pathway

**Figure 5:**
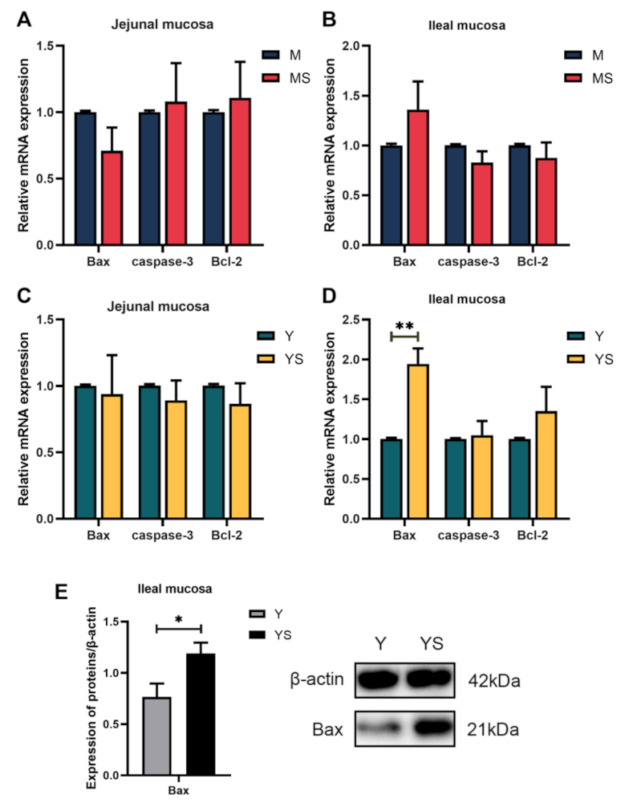
Effects of chronic cold stress on the expression of key genes and proteins in the apoptotic pathway of pig intestinal mucosa. (**A**,**B**) The mRNA expression of key genes of apoptosis in intestinal mucosa of Min pigs (n = 6). (**C**,**D**) The mRNA expression of key genes of apoptosis in intestinal mucosa of Yorkshire pigs (n = 6). (**E**) The protein expression level of Bax in the ileal mucosa of Yorkshire pigs (n = 6). Data are presented as the means ± SEM. * *p <* 0.05, ** *p <* 0.01.

The influence of chronic cold stress on intestinal apoptosis is shown in Figure 5. Compared with the M group, chronic cold stress had no significant effect on the mRNA expressions of Bax, caspase-3, and Bcl-2 in the jejunal and ileal mucosa of the MS group (*p* *>* 0.05, Figure 5A,B). Nevertheless, the mRNA and protein expression levels of Bax in the ileal mucosa of the YS group were significantly increased compared with the Y group (*p* *<* 0.05, Figure 5D,E).

### 2.5. Glucose Supplemented Diet Can Appropriately Alleviate Intestinal Injury Caused by Chronic Cold Stress

**Figure 6:**
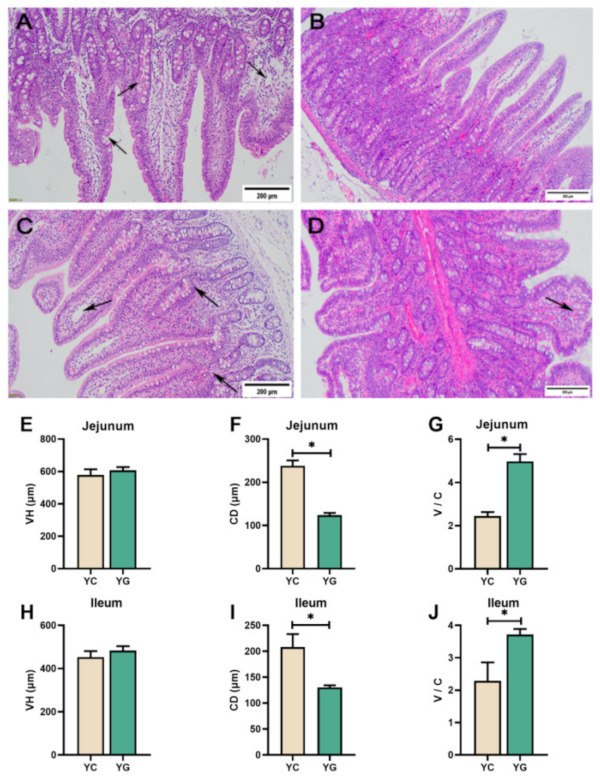
Effect of glucose-supplemented diet on jejunal and ileal slices of pigs under chronic cold stress. (**A**) In the YC group, there was a decrease in cupped cells in the jejunal mucosa and a goblet crypt with mucosal edema. (**B**) No significant abnormalities were seen in the jejunal mucosa of the YG group. (**C**) Local inflammatory cell infiltration, mucosal edema, and crypt enlargement in the ileal mucosa of the YC group. (**D**) No obvious impairment of ileal mucosa in the YG group. (**E**–**G**) VH, CD, and V/C in jejunum of the YC group. (**H**–**J**) VH, CD, and V/C in ileum of the YG group. Black arrows indicate abnormal mucosal development. Data are presented as the means ± SEM. * *p* *<* 0.05.

As shown in Figure 6, the jejunal mucosa in the Yorkshire control group under chronic cold stress (YC group) showed punctate epithelial disruption, enlarged crypt, reduced goblet cells, and mild mucosal edema (Figure 6A). The YC group’s ileal mucosa displayed local inflammatory cell infiltration, mucosal edema, as well as an expanded crypt (Figure 6C). In the Yorkshire pigs with glucose supplemented diet under chronic cold stress group (YG group), the mucosa of the jejunal mucosa remained intact, and the villi morphology and crypt were essentially normal, with occasional inflammatory cell infiltration (Figure 6B). The YG group showed mild edema in the ileal mucosa, and the rest was normal (Figure 6D). Under the effect of chronic cold stress, the CD of both jejunal mucosa and ileal mucosa was significantly decreased in the YG group compared with the YC group (*p* *<* 0.05, Figure 6F,I), while the V/C of jejunal mucosa and ileal mucosa was elevated in the YG group (*p* *<* 0.05, Figure 6G,J).

**Figure 7:**
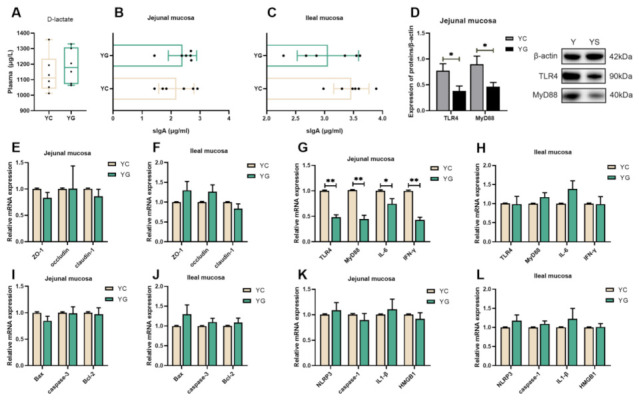
Effects of a glucose-supplemented diet on intestinal damage induced by chronic cold stress. (**A**) The level of D-lactate in the plasma of Yorkshire pigs (n = 6). (**B**,**C**) The content of sIgA in the intestinal mucosa of Yorkshire pigs (n = 6). (**D**) The proteins expression levels of TLR4 and MyD88 in the jejunal mucosa of Yorkshire pigs (n = 6). (**E**,**F**) The mRNA expression levels of tight-junction proteins in the intestinal mucosa of Yorkshire pigs (n = 6). (**G**,**H**) The mRNA expression of TLR4, MyD88, and inflammatory factor in the intestinal mucosa of Yorkshire pigs (n = 6). (**I**,**J**) The mRNA expression of key genes of apoptosis in the intestinal mucosa of Yorkshire pigs (n = 6). (**K**,**L**) The mRNA expression of key genes of pyroptosis in the intestinal mucosa of Yorkshire pigs (n = 6). Data are presented as the means ± SEM. * *p <* 0.05, ** *p <* 0.01.

Compared with the YC group, there were no changes in the plasma D-lactate content and the sIgA levels in the jejunal and ileal mucosa of the YG group, and the tight junction proteins (ZO-1, Occludin and claudin-1), pyroptosis, and apoptosis-related gene mRNA expression levels had no significant changes (*p* *>* 0.05, Figure 7). Notably, we found that the mRNA and protein expression of TLR4 and MyD88 in jejunum mucosa in the YG group were down-regulated compared with the YC group (*p* *<* 0.05, Figure 7D,G). Subsequently, it was found that the mRNA expression of IL-6 and IFN- γ in jejunal mucosa of the YG group was also lower than that of the YC group (*p* *<* 0.05, Figure 7G).

## 3. Discussion

The survival of mammals is inevitably threatened by external stressors. To acclimatize to the mutations of the surrounding environment, animals produce a series of physiological and biochemical reactions to sustain the balance of the body’s internal milieu [24]. Endotherms are easily stressed by chronic cold stress [25]. The healthy physiological function and development of the small intestine is critical for growth. When the function of the small intestine is compromised, the animal’s growth is hampered [26]. It has been previously noted that disordered villi and inflammatory cell infiltration were observed in the duodenum of quail subjected to cold stress [27]. After hypoxic-ischemic cold stress, Wistar rat pups’ small intestine mucosa showed severe separation of the submucosa from the lamina propria, necrosis, and loss of villi structure [28]. Muscle edema of the ileum and necrosis of the villi with partial loss of epithelial structures were observable in premature neonatal rats subjected to cold stress and hypoxia [29]. In our study, similar results were noticed in Yorkshire pigs; it was evident that the jejunal and ileal mucosa of the YS group had suffered different degrees of impairment compared with the Y group. In contrast, in Min pigs, no equivalent results were found. Intestinal VH and CD can reflect the development of the intestinal tract. Generally, higher VH and shorter CD indicate better intestinal development [30]. Previous studies have shown that cold stress at 2–8 °C can decrease VH, CD, and VH / CD in the intestines of broiler chicken [31]. In the study of piglets, it was also found that cold stress can reduce VH and VH / CD in the duodenum of piglets, and an increase in CD [32]. Furthermore, cold stress could also reduce the proliferation rate of intestinal epithelial cells in rats [33]. In this study, when compared with the Y group, chronic cold stress significantly down-regulated VH and V/C in the jejunum as well as V/C in the ileum in the YS group. In Min pigs, chronic cold stress had no effect on the growth of intestinal VH and CD. This indicates that prolonged cold stress impedes jejunal and ileal development in Yorkshire pigs, whereas Min pigs are unaffected by the negative effects of prolonged cold.

Tight-junction protein is the key to control intestinal permeability. It can hinder complex microorganisms and macromolecular metabolites from entering the body [34], and selectively allow the absorption of small molecular nutrients and the secretion of body fluids. Previous studies have shown that acute cold stress could up-regulate the mRNA expression of Occludin and ZO-1 in the jejunum of broilers, which may represent tight-junction reshaping [35]. Studies on heat stress have shown that the up-regulation of Occludin mRNA expression is an important defensive response to alleviate tight-junction injury induced by heat stress [36]. Interestingly, in our study, the mRNA and protein expression of Occludin in the jejunal mucosa of the YS group dropped compared with the Y group. These results illustrated that the jejunal mucosa, but not the ileal mucosa, of Yorkshire pigs was negatively affected by chronic cold stress, resulting in the damage of tight junctions. The tight-junction proteins in the intestinal mucosa of Min pigs were not affected by cold stress. These results implied that Min pigs have enough cold acclimation in the growth and development of the small intestine. Antimicrobial peptides (AMPs) are important effector molecules involved in innate immune responses in the small intestine [8]. PMAP-37, as one of the key natural antimicrobial peptides in mammals, has been proved to inhibit the growth of numerous harmful bacteria [37]. We observed that the relative expression of PAMP-37 mRNA in the jejunal mucosa of the YS group was reduced, which was the first evidence that cold stress caused PAMP-37 transcription to decrease in the small intestine. Nevertheless, there was no similar result in the ileal mucosa of the YS group. These results demonstrated that chronic cold stress had negative impacts on the homeostasis of the jejunal mucosa, while the intestinal function of the ileal mucosa seemed to be unchanged.

Elevated plasma D-lactate levels reflect the increased degree of small intestinal mucosal injury [38]. We found that the activity of D-lactate in plasma increased significantly in the YS group, indicating that chronic cold stress caused the injury of the intestinal mucosa of Yorkshire pigs. sIgA plays an irreplaceable role in the intestinal mucosal immune system [39]. As the crucial immunoglobulin on the small intestinal mucosa, sIgA is the first line of defense on the intestinal mucosa and has resistance to multiple endogenous commensal bacteria and foreign invading pathogens. Previous research has shown that cold can reduce the concentration of sIgA in the gut [40]. We reached a similar result that chronic cold stress resulted in a decline in sIgA concentrations in the jejunal mucosa of Yorkshire pigs. Decreased concentrations of sIgA indicated suppression of intestinal mucosal immunity, which made the jejunum more susceptible to pathogen invasion. However, there was no variation in the concentration of sIgA in the ileal mucosa of Yorkshire pigs, which indicated that the immune system of the jejunal mucosa was more severely impaired under chronic cold stress when compared with the ileal mucosa. IL-6 has been widely recognized as a pro-inflammatory factor [41]. The anti-inflammatory cytokines IL-4 and IL-10 are currently considered inflammatory and immunosuppressive factors [42]. In our research, the IL-6 mRNA expression in the jejunal mucosa of the YS group was intensified. Moreover, the IL-4 and IL-10 mRNA expression was reduced in the jejunal mucosa of the YS group. Whereas, it was not observed that these alterations were detected in the ileal mucosa of Yorkshire pigs. Surprisingly, this phenomenon was not observed in the MS group. Collectively, these data revealed that chronic cold stress increased the risk of inflammation in the jejunal mucosa of Yorkshire pigs, and the intestinal mucosa of Min pigs was not abnormally affected by chronic cold stress.

The TLRs signaling pathway plays a vital role in immune system reactions, and TLR4 can induce the expression of inflammatory-related genes through MyD88 [13]. Under external stress, the interaction of the TLR4 and the TIR domains of MyD88 provokes downstream signaling cascades that in turn lead to enlivening of NF-κB, which then activates the transcription of pro-IL-1β and NLRP3 genes [43]. Chronic cold stress was reported to activate the expression of TLR4 and MyD88 in livers of mouse [44]. In this study, we found that the mRNA and protein expressions of TLR4 and MyD88 in the jejunal mucosa of Yorkshire pigs were up-regulated. HMGB1, as a novel type of pro-inflammatory cytokine, is released into the extracellular fluid and interacts with TLR4 to activate NF-κB [45]. Meanwhile, HMGB1 is also a signal of pyroptosis [46]. Of particular interest, we observed that mRNA and protein levels of HMGB1 in jejunum mucosa in the YS group were increased. This insinuated that the intestinal mucosal pyroptosis pathway in Yorkshire pigs might be activated by chronic cold stress. Pyroptosis belongs to a different form of inflammatory programmed cell death. Upon sensing specific pathogen-associated molecular patterns (PAMPs) or damage-associated molecular-pattern molecules (DAMPs), such as particles, crystals, and ATP, NLRP3 recruit the adaptor protein apoptosis-associated speck-like protein containing a CARD (ASC) [47]. ASC couples the upstream NLRP3 to caspase-1 and caspase-1 is activated into cleaved caspase-1. Cleaved caspase-1 can then proteolytically cleave pro-IL-1β and pro-IL-18 into their respective mature forms [48]. It also promotes the cleavage and activation of gasdermin D, oligomerizes the N-terminal domain of gasdermin D, forms pores in the cell membrane, and results in cell swelling and release of cytoplasmic contents such as mature-IL-1β and mature-IL-18, which ultimately leads to the generation of inflammation in the body [47]. Our study showed that the NLRP3 mRNA and protein was elevated in the jejunal mucosa of the YS group. Furthermore, the caspase 1 and IL-1β mRNA was raised, and the cleaved caspase-1 and mature-IL-1β protein in the jejunal mucosa of the YS group were also increased. These suggested that chronic cold stress activated pyroptosis and induced inflammation in the jejunal mucosa of Yorkshire pigs. Consequently, the TLR4/MyD88 signaling pathway and pyroptosis may be important pathways for the induction of inflammation in the intestinal mucosa of Yorkshire pigs under chronic cold stress.

Apoptosis is the core factor in maintaining homeostasis in mammals, and cells in the body sustain homeostasis through equilibrium between cell proliferation and cell death [49]. When cell proliferation or cell death is incorrectly promoted, the development of tumors, neurodegenerative diseases, and autoimmune diseases was incited easily [13]. The Bcl-2 protein family controls and regulates mitochondrial apoptosis pathways [50]. This pathway can promote mitochondrial outer membrane permeabilization (MOMP) [51]. MOMP is generally considered to be an irreversible point in the apoptotic pathway. Members of the Bcl-2 family of proteins are classified as anti-apoptotic (e.g., Bcl-2), pro-apoptotic (e.g., Bax), and the Bcl-2 homology 3 (BH-3)-only initiators that respond to apoptotic stimuli [13]. Normally, the anti-apoptotic Bcl-2 protein antagonizes the pro-apoptotic Bax protein, and thereby restrained MOMP and hindered the activation of the mitochondrial apoptotic pathway [52]. Bcl-XL can mediate the reverse transport of pro-apoptotic protein Bax from mitochondria to the cytoplasm, thus preventing a plethora of Bax accumulation in the mitochondrial outer membrane (MOM) [53]. When cells are stimulated by pro-apoptotic factors, the oligomerization of Bax and Bak are induced to form pores in the mitochondrial outer membrane, and then cytochrome C is released in the mitochondria into the cytoplasm, which activates the caspase cascade and leads to apoptosis [54]. This is considered a critical step leading to apoptosis. Caspase-3 is an important protein in the apoptotic enzyme cascade. Bax can activate caspase-3 to indirectly inhibit Bcl-2 expression and induce apoptosis [55]. In our study, we observed strengthened mRNA and protein levels of Bax in the ileal mucosa of Yorkshire pigs under chronic cold stress. This suggested that the apoptotic pathway in the ileal mucosa of Yorkshire pigs is activated by chronic cold stress. IFN-γ is a cellular inflammatory factor in helping maintain homeostasis and immune and inflammatory responses. It has been reported that IFN-γ and TNF-α synergistically enhances the level of iNOS, resulting in the increase of endogenous NO, which in turn reduces the level of Bcl-2, increases the content of Bax, and leads to apoptosis of mouse cells [56]. Our experimental results showed that the mRNA and protein levels of IFN-γ were augmented in the ileal mucosa of the YS group. However, only the mRNA level was increased in the jejunal mucosa. These might be some of the reasons why the apoptosis pathway was promoted in the ileum mucosa of the YS group. In the MS group, the expression of associated genes did not change significantly, suggesting that continuous cold stress does not disturb the apoptotic equilibrium in the small intestinal mucosa.

Mammals subjected to acute cold stress maintain homeostasis by increasing glucose absorption in the body [57], while cold-adapted animals still demonstrate a considerable increase in glucose uptake in tissue [58]. It is self-evident that glucose is exceedingly critical for animals exposed to cold. Thus, we sought to determine whether supplementation of glucose during feeding could alleviate intestinal damage in pigs caused by chronic cold stress. Intriguingly, we observed that glucose alleviated the adverse effects of cold on the intestine by ameliorating the CD and V/C of the jejunal mucosa and ileal mucosa. In our research, we found that the TLR4 and MyD88 mRNA and protein in the jejunal mucosa of the YG group dropped, and the mRNA levels of IL-6 and IFN-γ were also reduced. This indicated that the addition of glucose in the feed suppresses the activation of the TLR4 signaling pathway and inflammatory factors in the jejunal mucosa induced by chronic cold stress, even though there was no effect on the apoptosis and apoptosis pathways. Furthermore, we discovered that plasma D-lactate had no effect on the level of sIgA in the intestinal mucosa. From the above, we speculate that the activation of pyroptosis and apoptotic pathways may have other factors and that these factors are not affected by dietary glucose supplementation.

**Figure 8:**
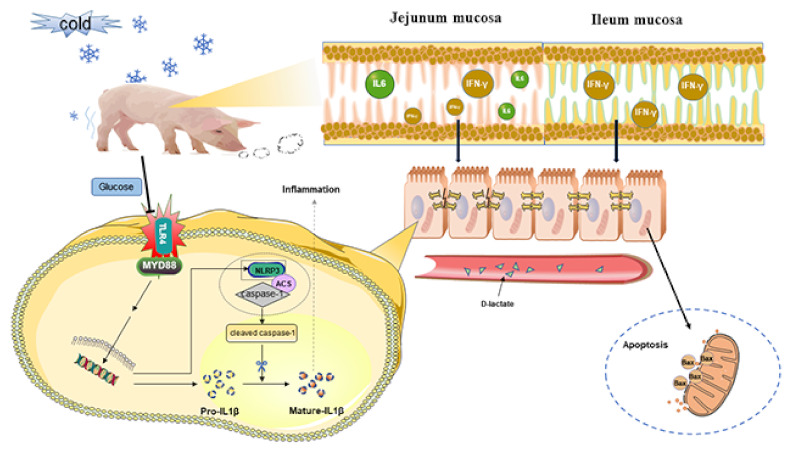
Cold stress induced small intestinal mucosal injury via TLR4/MyD88 signaling, and glucose supplementation alleviated small intestinal mucosal injury.

In conclusion, chronic cold stress did not adversely affect the small intestinal mucosa in the Min pig model. However, the intestinal barrier of jejunum mucosa was damaged in the Yorkshire pig model, and the TLR4 signaling pathway and pyroptosis were activated via chronic cold stress. Simultaneously, the apoptosis pathway of ileum mucosa was promoted by chronic cold stress. Furthermore, dietary glucose supplementation could reduce the activation of the TLR4 pathway induced by cold stress (Figure 8). This study provides new evidence for the negative effects of chronic cold stress on intestinal mucosal immune homeostasis, and provides new insights into ameliorating intestinal injury during chronic cold stress.

## 4. Materials and Methods

### 4.1. Animals

#### 4.1.1. Chronic Cold Stress Experiment

Two experiments were conducted individually. In experiment one, 12 Min pigs were randomly selected and separated into Min control group (M, basal diet, females, 35.52 kg ± 1.18 kg, 21 d, 17 ± 3 °C, maintain temperature with electronic heater, n = 6) and Min cold stress group (MS, basal diet, females, 35.48 kg ± 1.29 kg, 21 d, 7 ± 3 °C, consistent with the ambient temperature, n = 6). Then, a second experiment was conducted at the same time. In experiment two, 12 Yorkshire pigs were chosen at random and separated into two groups: Yorkshire control group (Y, basal diet, females, 24.83 kg ± 0.64 kg, 21 d, 17 ± 3 °C, maintain temperature with electronic heater, n = 6) and Yorkshire cold stress group (YS, basal diet, females, 24.80 kg ± 0.61 kg, 21 d, 7 ± 3 °C consistent with the ambient temperature, n = 6).

#### 4.1.2. Glucose Supplementation in the Diet of Chronic Cold Stress Pigs Experiment

The feeder animals were then fed a basic diet and a glucose supplemented diet simultaneously in a third experiment that was conducted in a prolonged cold exposure condition. Yorkshire pig models with chronic cold exposure were established in experiment three: 12 Yorkshire pigs were randomly divided into the Yorkshire cold stress control group (YC, basal diet, females, 23.54 kg ± 0.84 kg, 22 d, 8 ± 3 °C, consistent with the ambient temperature, n = 6) and Yorkshire cold stress glucose-supplemented diet group (YG, glucose-supplemented diet, females, 23.76 kg ± 0.78 kg, 22 d, 8 ± 3 °C, consistent with the ambient temperature, n = 6). Significantly, in comparison with the YC group, the food in the YG group was supplemented with glucose solely in the composition of the base diet formulation. Environmental temperatures during the study were monitored using a GSM501 wireless thermohygrometer (Guangzhou Rongce Electronics, Guangdong, China).

Each pig was individually housed in a steel metabolic cage. The metabolic cages were each equipped with an automatic feeder and a drinking fountain. The pigs were allowed free access to water and food. Before the experiment, use the potassium permanganate fumigation method was used to disinfect the pig house. After that, the ventilation 4 angel formaldehyde was cleared. Lime powder was provided at the entrance of the pig house. The feeding environment was sterilized every week. The experiment was carried out at Acheng Experimental Base of Northeast Agricultural University. Diets were formulated to meet the nutrient requirements of piglets (MOA, 2020 and NRC, 2012). The nutrient levels and composition of diet were shown in Table 1 and Table 2.

The protocols of this study were authorized by the Northeast Agricultural University Institutional Animal Care and Use Committee (NEAU—[2011]—9), and the animal care and treatment procedures satisfied the requirements of Heilongjiang Province’s “Laboratory Animal Management Regulations” (updated 2016).

### 4.2. Collection of Experimental Samples

On day 22 of the first and second experiments and on day 23 of the third experiment, all pigs from each group were euthanized. Before slaughter, 10 mL of blood was drawn from each pig’s vein, put in a heparin sodium anticoagulant tube, centrifuged for 15 min at 300× *g* (4 °C) to collect plasma, and stored at −40 °C. Mucosa scrapings from intestine segments, including jejunum and ileum, were harvested for analysis of cytokine, immunoglobulin, protein, and gene expression. The samples were stored at −80 °C until use, except for the samples for histological analysis, which were assayed immediately after sampling.

### 4.3. Measurement of Intestinal Morphology

The jejunum and ileum tissues were washed separately in saline before being fixed in 10% neutral formalin. The fixed samples were dehydrated in alcohol, embedded in paraffin, and sectioned, with hematoxylin and eosin staining applied afterward [29]. The morphological structure was observed under a light microscope. VH, CD, and V/C of jejunum and ileum were measured by image-Pro Plus4.5.1 (Media Cybernetics Inc., Bethesda, MD, USA).

### 4.4. Intestinal Permeability Markers

Determination of plasma D-lactate concentration using ELISA kit as per manufacturer guidelines of the kit (Shanghai Hengyuan Biological, Shanghai, China).

### 4.5. Detection of Intestinal Inflammation and Immune Factors

For the measurement of intestinal inflammation and immune factors concentrations, a 10% tissue homogenate of jejunal and ileal mucosa was produced using saline [59]. The total protein concentration was calculated using the BCA Protein Assay Kit (Beyotime Biotechnology, Nantong, China). The concentrations of IFN-γ and sIgA in jejunal mucosa and ileal mucosa were measured using an ELISA kit per the manufacturer’s instructions (Shanghai Hengyuan Biological, Shanghai, China).

### 4.6. Real-Time Polymerase Chain Reaction Analysis

Total RNA was extracted from jejunal mucosa and ileal mucosa using TRIZOL reagent (Thermo Fisher Scientific Co., Ltd., Shanghai, China), and then the concentration of RNA was measured using a nanophotometer (MmilenGmbH, Munich, Germany), and the A260/A280 ratio was kept around 1.8 and 2.0. Total RNA was reverse transcribed using a 5X Integrated RT MasterMix (Dining, Beijing, China). An ABI 7500 Fast Real-Time PCR System (Foster City, CA, USA) was used to perform 40 cycles of RT-PCR, with each cycle consisting of 95 °C for 30 s, 95 °C for 5 s, and 60 °C for 30 s. The total reaction volume was 20 μL, with 1 μL of cDNA, 7 μL of 0.1 percent DEPC water, 10 μL of 2 Fast qPCR Master Mixture (Dining, Beijing, China), and 1 μL each of upstream and downstream primers. Each relative target gene level was calculated using β-actin as a control. At least two replicates were used in each response. Data were calculated using the 2^−ΔΔCt^ method [60]. The amount of detected RNA was normalized to the amount of β-actin RNA. Information on the primers involved in the experiment can be found in Table S1.

### 4.7. Western Blot Analysis

Total proteins were extracted from jejunal and ileal mucosa using RIPA Lysis Buffer (Beyotime Biotechnology, Shanghai, China) complemented with PMSF (Beyotime Biotechnology, Shanghai, China), and total protein concentration was gauged using a BCA Protein Assay kit (Beyotime Biotechnology, Shanghai, China). After SDS-PAGE, the corresponding gel bands were meticulously cut per the molecular weight of the target protein and carefully transferred to a polyvinylidene fluoride (PVDF) membrane. The PVDF membrane was placed in a solution containing 5% nonfat dry milk in TBST and placed on a shaker at 35 °C for two hours. After that, the PVDF membranes were removed and placed in primary antibody dilutions containing primary antibodies, which were then left to stand at 4 °C for 12 h. After 12 h of incubation, the PVDF membranes were removed and washed three times with 1 × TBST for 15 min each. The membranes were then incubated for 2 h at room temperature with [HRP Goat Anti-Rabbit IgG (H + L)] before being washed three times with 1 × TBST for 15 min each time [8]. Finally, the proteins on the strips were visualized using the BeyoECL Star Fluorescence Detection Kit (Beyotime Biotechnology, Shanghai, China). A gel imaging and analysis system was used to look for protein bands (UVItec, Cambridge, UK). Image J software was used to calculate protein intensity. Information about antibodies in this study is shown in Table S2.

### 4.8. Image Analysis and Data Statistics

The experimental data were sorted by Microsoft Excel 2016, and then analyzed using SPSS 25.0 (IBM-SPSS Inc, Chicago, IL, USA) for *t*-test. Visualization in this study was performed using GraphPad prism (version 8.0.2, Graph Pad Software Inc., San Diego, CA, USA). The statistical results are expressed by mean and standard error (SEM), and *p* *<* 0.05 was used as the criterion for judging the significance of difference.

## Figures and Tables

**Table 1:** Composition of experimental diets.

Basic Diet Ingredients	Content (%)
Corn	73.00
Soybean meal, de-hulled	15.30
Full-fat soybean meal, puffed	5.00
Fish meal	2.00
Soybean oil	1.00
L-Lysine	0.39
DL-Methionine	0.04
L-Threonine	0.12
L-Tryptophan	0.02
Calcium hydrogen phosphate	1.19
Limestone	0.66
Salt	0.28
Premix ^A^	1.00
**Nutrient levels ^B^**	
NE (Mcal/kg)	2.50
Crude protein	16.03
Lysine	0.98
Methionine	0.29
Threonine	0.60
Leucine	0.17
Calcium	0.66
Total phosphorus	0.56
Available phosphorus	0.33
Sodium	0.14
Chlorine	0.19

^A^ Provided the following per kilogram of diet: Fe, 160 mg; Cu, 150 mg; Mn, 40 mg; Zn, 140 mg; Se, 0.4 mg; I, 0.5 mg; vitamin A, 8000 IU; vitamin D3, 2000 IU; vitamin E, 30 mg; vitamin B1, 1.60 mg; vitamin B2, 5.00 mg; vitamin B6, 5.00 mg; vitamin B12, 0.01 mg; pantothenic acid, 20 mg; niacin, 15 mg; biotin, 0.05 mg. ^B^ Nutrient levels were calculated values.

**Table 2:** Composition of glucose-supplemented diets.

Basic Diet Ingredients	Content (%)
Corn	60.68
Soybean meal, de-hulled	17.53
Full-fat soybean meal, puffed	5.00
Fish meal	2.00
Glucose	10.00
Soybean oil	1.00
L-Lysine	0.35
DL-Methionine	0.05
L-Threonine	0.11
L-Tryptophan	0.01
Calcium hydrogen phosphate	1.25
Limestone	0.62
Salt	0.40
Premix ^A^	1.00
**Nutrient levels ^B^**	
NE (Mcal/kg)	2.63
Crude protein	16.03
Lysine	0.98
Methionine	0.29
Threonine	0.60
Leucine	0.17
Calcium	0.66
Total phosphorus	0.56
Available phosphorus	0.34
Sodium	0.19
Chlorine	0.26

^A^ Provided the following per kilogram of diet: Fe, 160 mg; Cu, 150 mg; Mn, 40 mg; Zn, 140 mg; Se, 0.4 mg; I, 0.5 mg; vitamin A, 8000 IU; vitamin D3, 2000 IU; vitamin E, 30 mg; vitamin B1, 1.60 mg; vitamin B2, 5.00 mg; vitamin B6, 5.00 mg; vitamin B12, 0.01 mg; pantothenic acid, 20 mg; niacin, 15 mg; biotin, 0.05 mg. ^B^ Nutrient levels were calculated values.

## Data Availability

The data presented in this study are available on request from the corresponding author. The data are not publicly available due to the fact that we are conducting further experiments.

## Supplementary Materials

The following supporting information can be downloaded at: https://www.mdpi.com/article/10.3390/ijms23147730/s1.
