# Supplementary material for: Dietary Glucose Ameliorates Impaired Intestinal Development and Immune Homeostasis Disorders Induced by Chronic Cold Stress in Pig Model
Source: Int J Mol Sci. 2022 Jul 13;23(14):7730. doi: 10.3390/ijms23147730 (PMC9317271; doi:10.3390/ijms23147730)
Supplement: Supplementary file 1 [file ijms-23-07730-s001.zip › Table S1.pdf]

Table S1 The real-time PCR primers

Gene	GenBank ID	Primer sequences (5' to 3')
β-actin	AY550069	F: ATGCTTCTAGGCGGACTGT
		R: CCATCCAACCGACTGCT
ZO-1	XM_005659811.1	F: AGCCATCCACTCCTGCCTAT
		R: GGGACCTGCTCATAACTTCG
Occludin	NM_001163647.2	F: CAGGTGCACCCTCCAGATTG
		R: ATGTCGTTGCTGGGTGCATA
Claudin-1	NM_001244539.1	F: GCCCTACTTTGCTGCTCCTG
		R: TTTCTGGTTGTTCCCACACG
MUC2	XM_021082584.1	F: TCCCAGGTCGAGTACATCCT
		R: CTATAGTGCGGGGGGTGCTGAC
EGF	NM_214020.2	F:ACCCTACTCCACCCTCTCAC
		R:GTCTCTGTGCTGACATCGCT
CDX2	NM_001278769.1	F:TGGTGTGAGGGAGTGTTTCG
		R:AAAGTCCAGGTTGGCTCTGG
PMAP-37	L39641.1	F: CTTAGCCGACTGCGTGAT
		R: TAGCCTGAATCTTAGGACTGAA
PR-39	L23825.1	F: GAACCCATCCATTCACTCAC
		R: TTATCAGCCACTCCATCACC
IL-6	NM_214399	F: ACAAAGCCACCACCCCTAAC
		R: CGTGGACGGCATCAATCTCA
INF-γ	NM_214304.1	F: ATGAGGCAGAGTACCGGGAT
		R: AGCGTAAGCATTGCGGATCT
TNF- α	NC_010449.5	F: TTGAGCATCAACCCTCTGGC
		R: GGCATACCCACTCTGCCATT
IL-4	NM_214123.1	F: CTCCCAACTGATCCCAACCC
		R: TGCACGAGTTCTTTCTCGCT
IL-10	NM_214041.1	F: CCTGGAAGACGTAATGCCGA
		R: CACGGCCTTGCTCTTGTTTT
TLR4	NM_001113039.2	F: CAGTCAAGATACTGGACCTGAGC
		R: GGCTCCCAGGGCTAAAACTCT
MyD88	NM_001099923.1	F: CCATTCGAGATGACCCCCTG
		R: TAGCAATGGACCAGACGCAG
NLRP3	NM_001256770.2	F: CTGGGACTCTGACTAGGGCT
		R: TTTTTCTGTCTGGCCCCGAG
Caspase1	NM_214162.1	F: TACAAGAATCCCAGGCGGTG
		R: CCTTTGGGCTATGTCTGGGG
IL-1β	NM_2140551.1	F: GCCAACGTGCAGTCTATGGAGTG
		R: GGTGGAGAGCCTTCAGCATGTG
HMGB1	NM_001004034.1	F: GAGGAAACTTGAGACCCACCA
		R: GTGTCCTTCCTTCCCTCATGT
Bax	XM_005664710	F: ATGGAGCTGCAGAGGATGAT
		R: AAAGTAGAAAAGCGCGACCA

Caspase3	NM_214131	F: CGGACAGTGGGACTGAAGTA<br>R: GATCCGTCCTTTGAATTTCG
Bcl2	NM_001164511.2	F: ACTTCTGCGAAAGCGAATTGCC<br>R: AGCCTCCGTTTTGCCTTATCC
