# Supplementary material for: Dietary Glucose Ameliorates Impaired Intestinal Development and Immune Homeostasis Disorders Induced by Chronic Cold Stress in Pig Model
Source: Int J Mol Sci. 2022 Jul 13;23(14):7730. doi: 10.3390/ijms23147730 (PMC9317271; doi:10.3390/ijms23147730)
Supplement: Supplementary file 1 [file ijms-23-07730-s001.zip › Table S2.pdf]

Table S2 Information for primary and secondary antibodies

Primary antibodies	Dilution ratio	Source of antibodies	Cat.No.	Clone type
Rabbit Anti-Occludin antibody	1：1000	ABclonal, Wuhan, China	A2601	Polyclonal antibody
Rabbit Anti-NLRP3 antibody	1：1000	Wanleibio, Shenyang, China	WL02635	Polyclonal antibody
Rabbit Anti-cleaved caspase-1 antibody	1：1000	Wanleibio, Shenyang, China	WL03450	Polyclonal antibody
Rabbit Anti-Bax antibody	1：1000	Bimake，Houston，TX，USA	A5131	Polyclonal antibody
Rabbit Anti-TLR4 antibody	1：1000	Bioss, Beijing, China	bs-20594R	Polyclonal antibody
Rabbit Anti-mature-IL-1β antibody	1：1000	Wanleibio, Shenyang, China	WL00891	Polyclonal antibody
Rabbit Anti-MyD88 antibody	1：1000	Bimake，Houston，TX，USA	A5440	Polyclonal antibody
Rabbit Anti-HMGB 1 antibody	1：1000	Wanleibio, Shenyang, China	WL03023	Polyclonal antibody
β-Actin Rabbit mAb	1：50000	ABclonal, Wuhan, China	AC038	Polyclonal antibody

Secondary antibody	Dilution ratio	Source of antibodies	Cat.No.
HRP Goat Anti-Rabbit IgG (H+L)	1：5000	ABclonal, Wuhan, China	AS014
